# Type 2 diabetes genetic association database manually curated for the study design and odds ratio

**DOI:** 10.1186/1472-6947-10-76

**Published:** 2010-12-30

**Authors:** Ji Eun Lim, Kyung-Won Hong, Hyun-Seok Jin, Yang Seok Kim, Hun Kuk Park, Bermseok Oh

**Affiliations:** 1 Department of Biomedical Engineering, School of Medicine, Kyung Hee University, Seoul, Korea; 2 Department of Physiology College of Oriental Medicine, Kyung Hee University, Seoul, Korea

## Abstract

**Background:**

The prevalence of type 2 diabetes has reached epidemic proportions worldwide, and the incidence of life-threatening complications of diabetes through continued exposure of tissues to high glucose levels is increasing. Advances in genotyping technology have increased the scale and accuracy of the genotype data so that an association genetic study has expanded enormously. Consequently, it is difficult to search the published association data efficiently, and several databases on the association results have been constructed, but these databases have their limitations to researchers: some providing only genome-wide association data, some not focused on the association but more on the integrative data, and some are not user-friendly. In this study, a user-friend database of type 2 diabetes genetic association of manually curated information was constructed.

**Description:**

The list of publications used in this study was collected from the HuGE Navigator, which is an online database of published genome epidemiology literature. Because type 2 diabetes genetic association database (T2DGADB) aims to provide specialized information on the genetic risk factors involved in the development of type 2 diabetes, 701 of the 1,771 publications in the type 2 Diabetes case-control study for the development of the disease were extracted.

**Conclusions:**

In the database, the association results were grouped as either positive or negative. The gene and SNP names were replaced with gene symbols and rsSNP numbers, the association p-values were determined manually, and the results are displayed by graphs and tables. In addition, the study design in publications, such as the population type and size are described. This database can be used for research purposes, such as an association and functional study of type 2 diabetes related genes, and as a primary genetic resource to construct a diabetes risk test in the preparation of personalized medicine in the future.

## Background

The prevalence of type 2 diabetes has reached epidemic proportions worldwide with the largest increase in Asia, Africa and South America [1]. The incidence of life-threatening complications of diabetes, such as retinopathy, nephropathy and lower-limb amputation, caused by the continued exposure of tissue to the high glucose has increased [2]. Since hyperglycemia can be prevented and reversed significantly by lifestyle changes, including the exercise and nutrition, diabetes risk tests, such as one provided by the American Diabetes Association http://www.diabetes.org, have been used to alarm various high risk groups. However, an increase in the incidence of diabetes has not been stopped over the last decade, highlighting the need for new approaches. According to the World Health Organization (WHO), the number of people with type 2 diabetes worldwide was approximately 170 million and 280 million in 2000 and 2010, respectively, which is expected to increase to 430 million by 2030 [1,3]

Following the growth of genomics, the disease susceptibility of human genetic variations has been examined to provide a better understanding of the pathophysiology of diabetes. The advances in genotyping technologies have increased the scale and accuracy of genotype data, thereby expanding enormously the number of genetic studies demonstrating a relationship between diseases and genetic variations. Consequently, it is difficult to search the massive number of publications from the text files in PubMed http://www.ncbi.nlm.nih.gov/pubmed, which researchers normally access to obtain information. Moreover, a systematic comparison of published data is not possible without considerable effort. Since association studies may have false positives or true negatives, it is important to compare one study with another before drawing a conclusion as to whether the association is true or not. Furthermore, the genetic effect size expressed by the odds ratio in association analysis is not always easy to find from text files.

With these problems in this field, considerable effort has been made to implement public genetic association databases. There are several genetic association databases, such as Genetic Association Database http://geneticassociationdb.nih.gov, dbGAP http://www.ncbi.nlm.nih.gov/sites/entrez?db=gap[4] from NCBI and Catalog of Published Genome-Wide Association Studies http://www.genome.gov/gwastudies[5] from NHGRI. The last two databases focus on the GWAS data, providing a list of genes and their association data as tables. Therefore, its value to researchers who want to examine the association studies carried out using the candidate gene approach has been reduced. Moreover, specific to diabetes, there are several databases, such as T2D-Db http://t2ddb.ibab.ac.in[6] and T1Dbase http://www.t1dbase.org[7]. These databases deal with genetic association studies as well as more integration resources involving gene expression, pathway and protein-protein interaction.

To provide focused information on a T2D association study, this study designed the T2D Genetic Association Database (T2DGADB). T2DGADB using 701 publications of the T2D study provides genetic association data that was manually curated and searchable. The web-based application displays comprehensive summaries of the published T2D genetic association results for browsing, visualization and mining. In addition, the data was displayed graphically adding convenience to understanding them.

## Construction and content

### Data collection

The list of publications used in this study was collected from the HuGE Navigator (version 1.3, http://hugenavigator.net) [8,9], which is an online database of published genome epidemiology literature. The Type 2 Diabetes Mellitus related articles were searched using the HuGE Navigator Phenopedia [10], which was developed to search gene-disease association summaries using the disease name as the search item. 1,771 published studies beginning from 2001 to October 5, 2009 were obtained from the search. Among them, only case-control study articles for Type 2 Diabetes (T2D) development were selected in order to exclude the articles relevant to diabetes complications (e.g., nephropathy, retinopathy), haplotype analysis and drug treatment (e.g., sulphonylurea, troglitazone, metformin). T2DGADB aims to provide specialized information on the genetic risk factors involved in the development of Type 2 diabetes. The articles on diabetes complications normally deal with the prognostic process of diabetes, and the articles on drug treatments deal with the pharmacogenetic aspects of diabetes patients. The decisions as to how the data on haplotype analysis would be collected are difficult because T2DGADB focused on information regarding each SNP association including the populations used, odds ratio etc. In this point, haplotype analysis could not be well fitted in the format. The final dataset was 701 publications in the Type 2 Diabetes case-control study for the development of the disease.

To select the 701 T2D development papers, the abstracts of 1,771 articles were downloaded through PubMed to be classified into T2D case-control articles or others. This information along with the title, authors, abstract, journal, online or in print publication date and PubMed ID to hyperlink to PubMed Abstract plus were deposited in the T2D Genetic Association Database. In addition, the full text of the 701 articles were downloaded, if accessible, to obtain all available text, tables, figures and supplemental data from the original articles. 625 of the 701 articles could be accessed but 76 articles could not be downloaded or were not written in English.

### Data incorporation

Since dbSNP builds and human genome builds were updated frequently from 2001 to 2009, it was important to change the Gene names, SNP positions and SNP rs number. Some articles reported only the SNP position and nucleotide change information (e.g., -4034A > C) instead of SNP ID (rs number), and in a few cases, they used their own SNP ID (e.g., SNP1, UCSNP-44). Old gene names and gene aliases were replaced with the Entrez gene official symbol (e.g., sAC→ADCY10, PC-1→ENPP1, last updated Aug 20, 2009).

The author's own SNP ID or SNP position information was replaced with the rs number using the HuGE Navigator Variant Name Mapper, which is an online tool to map the common variation names and rs numbers of genetic variants. However, it was too limited to find all the SNPs missing their own rs number because only 1,159 genes and 5,646 variants were deposited in the database (Aug 20, 2009). An alternative method was to use other databases, NCBI dbSNP, USCS Genome Browser and UCSC In-Silico PCR tool, based on the information provided by the articles. Some papers reported amino acid change information instead of the SNP ID. In this case, they were changed into rs numbers using SNP GeneView of NCBI dbSNP (e.g., PPARG, Pro12Ala→rs1801282). In some cases, a pair of PCR primer sequences was provided for the SNP. The UCSC In-Silico PCR tool can obtain a PCR product sequence with the primer sequences reported in the paper. The UCSC Genome Browser could confirm whether the locations of the PCR product sequences match the genes or chromosome locations described by the author, and the SNPs within the sequences were selected.

### Summary of the Data

**Figure 1:**
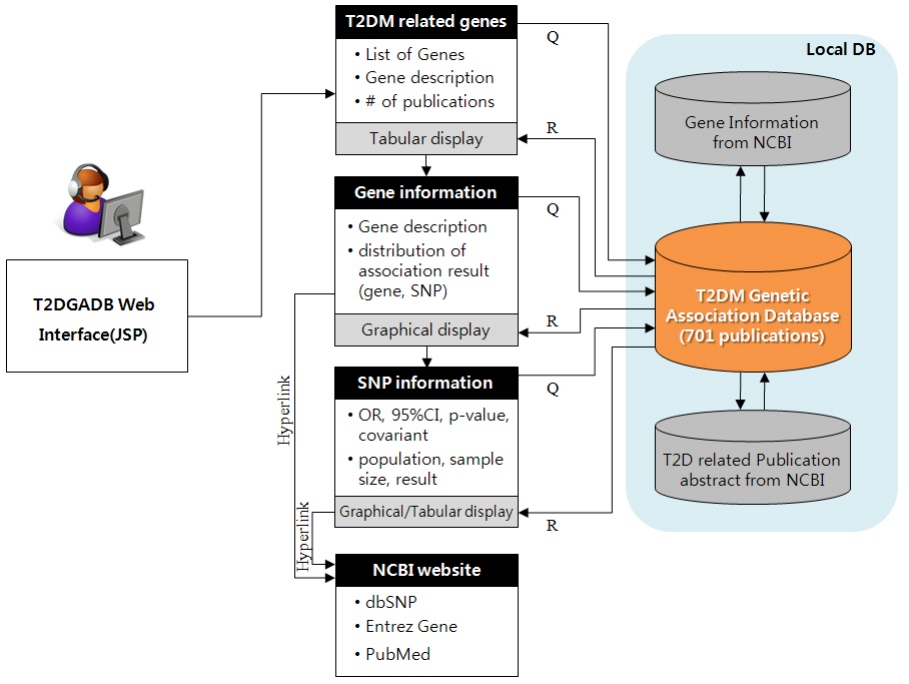
**Workflow through T2DGADB**.

T2DGADB contains information on the study design and SNP association result (Figure 1). The study design information summarizes the case and control sample size, sample population (e.g., Korean, Indian, American), age and gender of the subjects and study cohort data (e.g., Finland-U.S. Investigation of NIDDM Genetics (FUSION), Nurses' Health Study), if available.

SNP association information summarizes the results of an association study for each SNP or variant. It contains variation information, such as position information, common name of SNP as reported by the authors, dbSNP rs number, gene name, allele frequency of the reported allele, which is minor allele or risk allele, odds ratio and 95% confidence interval (CI) and p-value. Information on the covariants was added if the authors reported an adjusted p-value for the confounding factors. The association result was determined to be "associated" if the p-value was <0.05 or the odds ratio and 95% CI were in a suitable range. In some cases, the range of odds ratio and 95% CI was suitable but the association result was not associated because the adjusted p-value was not <0.05. In the case where both results were provided in the article, the adjusted p-value was used to determine the association.

### Web implementation and Database design

T2DGADB web interface was implemented in JSP, HTML and Javascript and run on CentOS (version 4.7) with the Apache-Tomcat (version 6.0.18) web server. MySQL (version 4.1.18) was used as the DBMS (Database Management System).

Figure 1 described the data workflow through T2DGADB. T2DGADB includes three local databases such as T2D genetic association database that summarise 701 publications, gene information database that is obtained from NCBI Entrez Gene database, and T2D related publication abstract database that is obtained from NCBI PubMed database. T2DGADB contains three main web pages such as total T2D gene list page, gene information page summarizing distribution of association results of gene and SNPs, and SNP information page summarizing study design and association results of SNPs. Figure 1 also illustrates that user can get the information from T2D related gene list page to SNP information page, sequentially. Each gene, SNP and publication is hyperlinked to NCBI Entrez gene, dbSNP and PubMed, respectively.

## Utility

### Common features

**Figure 2:**
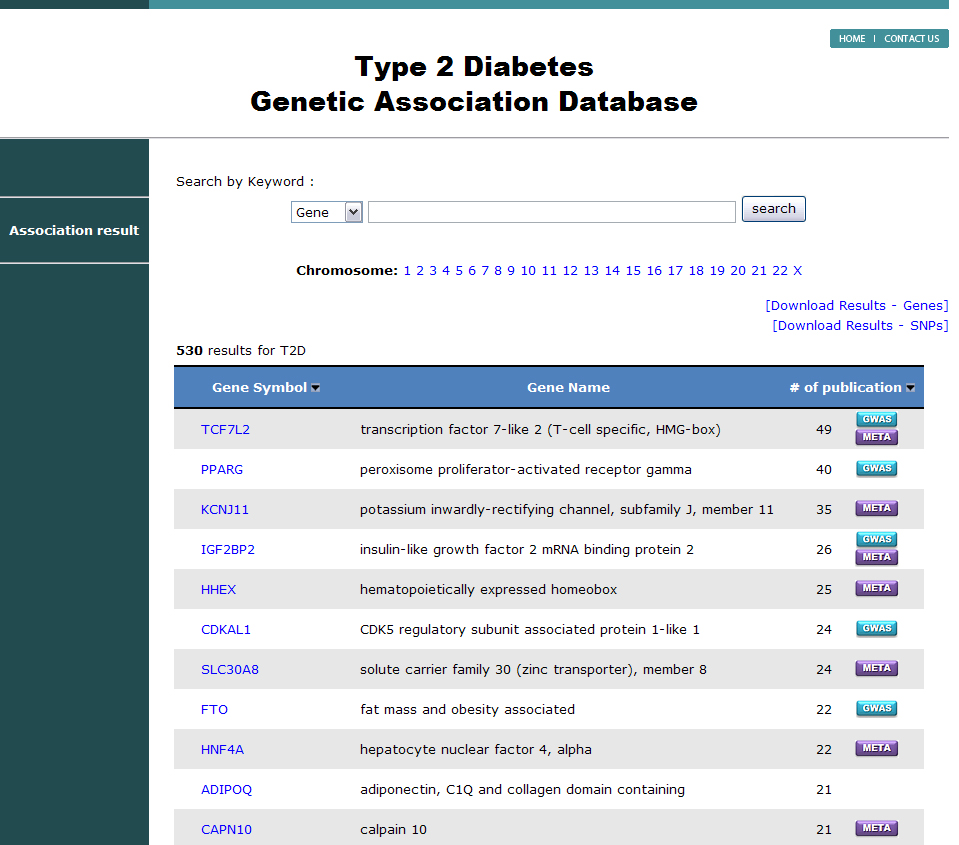
**Main page of the database**. Display of the genes that have been reported with Type 2 Diabetes and the number of publications.

T2DGADB provides a list of T2D associated genes, graphical views of their associations, variations that have been studied, links to the PubMed abstracts and more. Five hundred and thirty T2D gene lists, the full name of each gene and the number of published papers were found from the front page of the web site http://t2db.khu.ac.kr:8080 (Figure 2). The flags of "GWAS" and "META" in the publication column represent that the gene flagged includes the data from GWAS or meta-analysis. Search results in T2DGADB such as gene list, SNP rs number list and publication list are downloadable. The gene symbol, Entrez gene ID and official gene full name were referred to the Entrez gene record in GenBank. The location of chromosome band was presented instead of the gene name if the associated SNP was located between the gene and the gene in the intergenic region. However, the gene name for the SNP was used if the authors reported the genes that were nearest to the SNP. T2DGADB is a searchable web-based application that researchers can search using keywords such as the gene name, SNP rs number and chromosome.

### T2D gene information features

**Figure 3:**
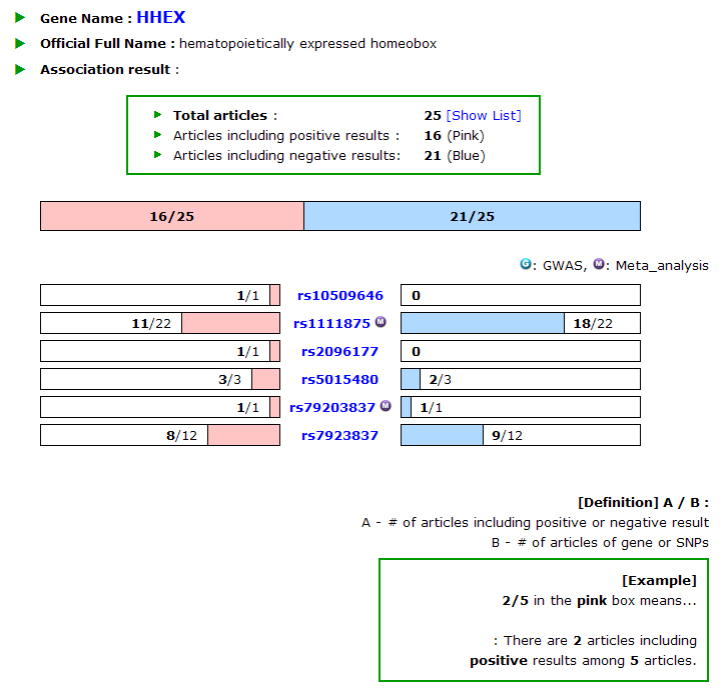
**Display of the distribution of the association result for HHEX and each SNP (Gene information features)**.

**Figure 4:**
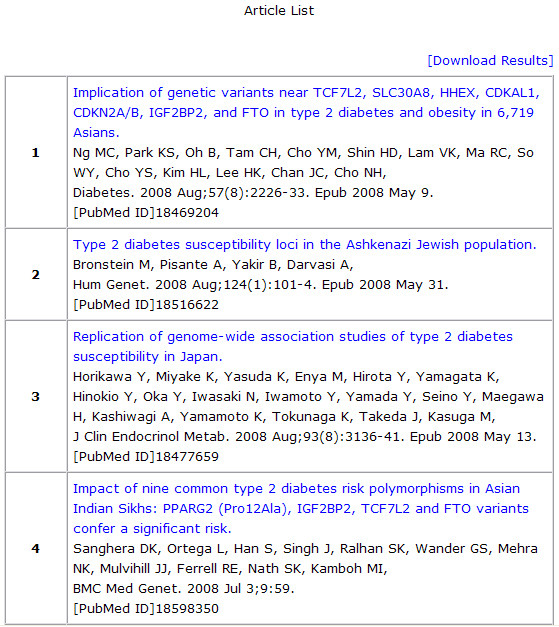
**Article list for HHEX as an example(Gene information features)**.

Searching by the gene name or clicking an interested gene in the front page leads to a T2D gene information page, offering the SNP and literature information of the gene. This page provides a bar graph of the association results as to whether the publication results are positive or negative in addition to the number of articles involved (Figure 3). A positive and negative result data is represented in red and blue color, respectively. One article might have more than one SNP analyzed in the study, and in some cases, each SNP may be examined with various samples in the paper. Therefore, the sum of the publication number for the positive and negative result might be higher than the total number of publications. For example, if one article deals with 2 SNPs of which one SNP is negative in the association result and the other is positive, it displays one negative and one positive result in the gene information page, even though there is only one publication. To prevent confusion, a list of articles containing the title, author and journal name, which appears by clicking the total articles, are also offered and the articles are hyperlinked to PubMed (Figure 4). The bar graph of each gene was drawn to scale according to the ratio of the number of positive to negative results. In the lower panel of the T2D gene information page, there are more bar graphs dedicated to the respective SNPs, which describe the positive and negative results by number. The gene symbol is linked to the Entrez Gene in NCBI http://www.ncbi.nlm.nih.gov/gene/, and clicking the rs number in the lower panel leads to the association results of the SNPs.

### SNP association result features

**Figure 5:**
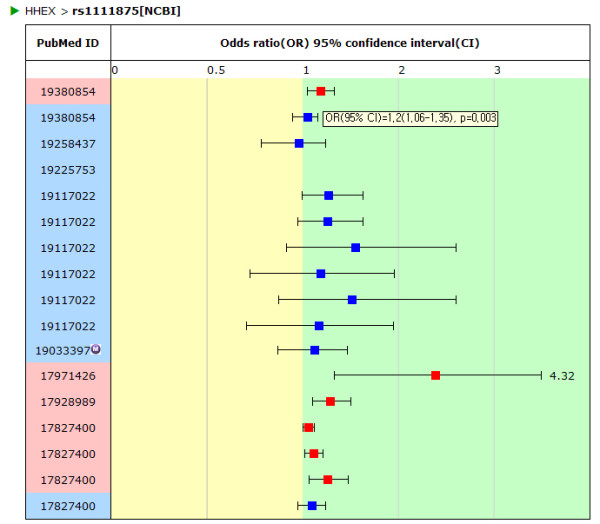
**Box plot (SNP association result features)**. The box plot graphs that include OR, 95% CI and p-value of rs1111875 as an example.

**Figure 6:**
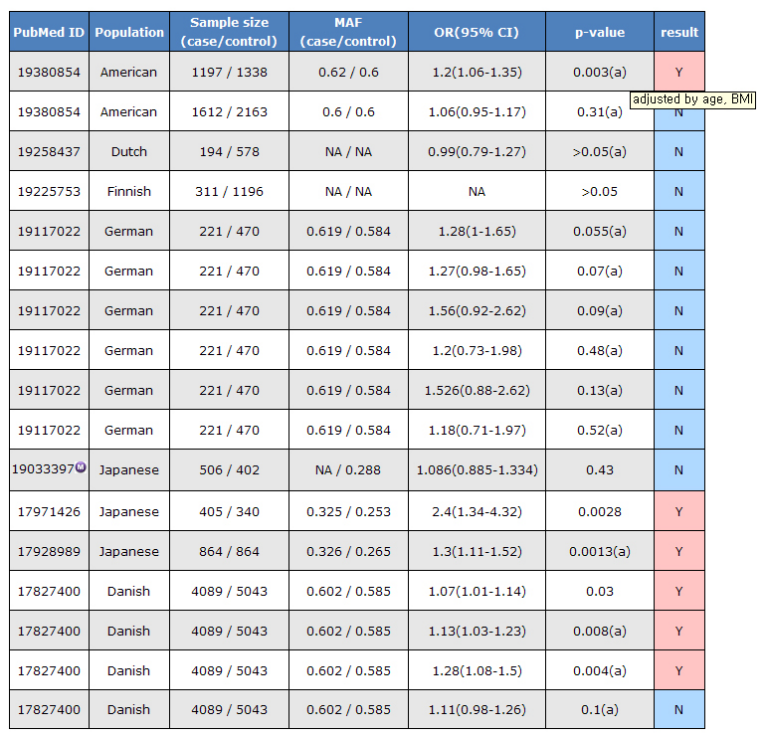
**Results table (SNP association result features)**. The table that includes the population, sample size, OR, 95% CI and p-value of rs1111875 as an example.

The SNP association result page provides T2D association-related information graphically, such as the OR, 95% CI and p-value (Figure 5). Each entry is displayed as a box plot. The red and blue color of the PubMed ID column and box of the box plot indicates a positive or negative association result, respectively. The box represents the OR and the horizontal bar indicates the boundary of the 95% CI. The association results were not presented in the graph when authors did not provide an OR or 95% CI. The SNP association result table shows detailed information on each article which includes the population, sample size, OR, p-value and more (Figure 6). It shows the best result of each SNP. Although the author reported a model-based OR and p-value, such as the additive, dominant, recessive, codominant model or logistic regression results, the most significant value among them is shown. If the p-value was obtained by adjusting the confounding factors in the article, they are marked with "(a)" and the list of confounding factors are provided. The adjusted p-value is selected if the article showed both the adjusted and crude p-value. For this reason, some data is represented as "no association" even when there is a proper OR and 95% CI. In the table, PubMed ID is linked to the article information data, which provides the title, abstract, authors and journal name.

### Analysis of T2DGADB data

**Figure 7:**
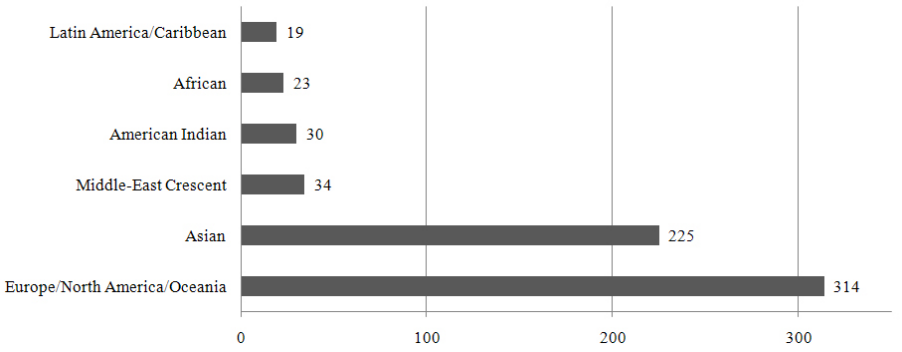
**Number of publications according to ethnic group**.

**Figure 8:**
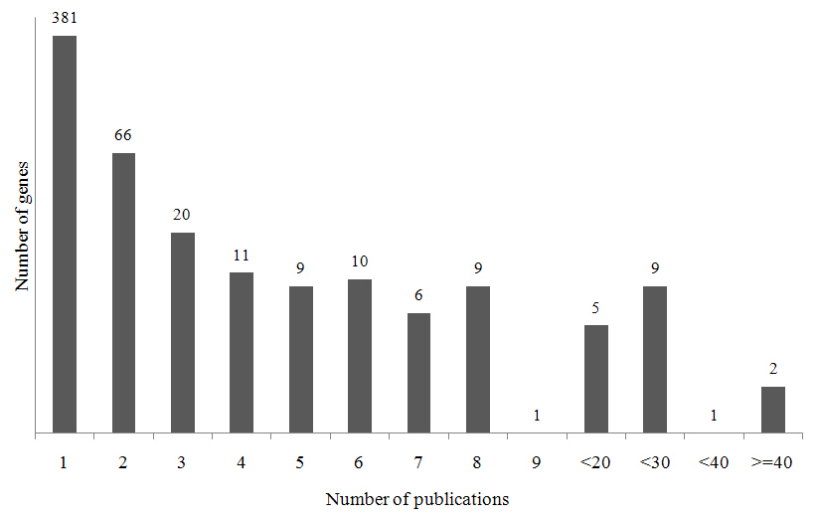
**Number of genes according to number of publications**.

**Figure 9:**
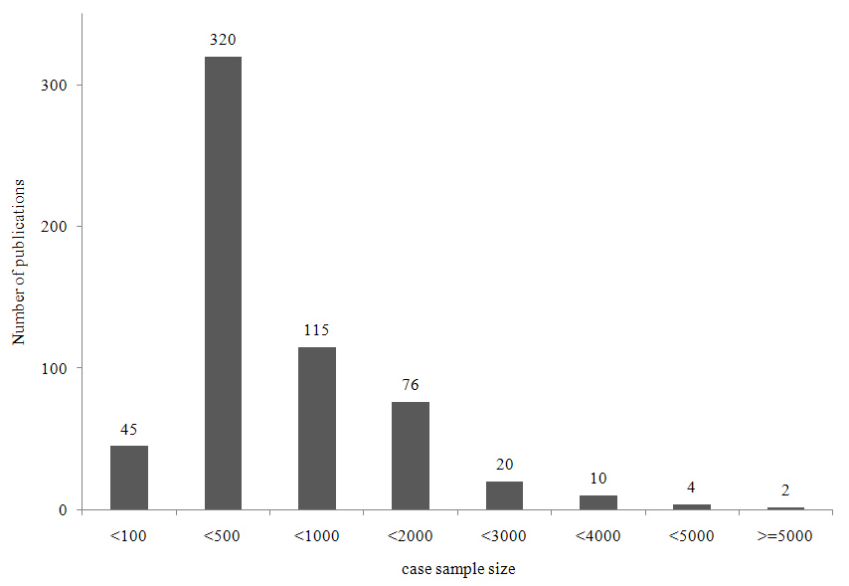
**Number of publications according to case sample size**.

**Figure 10:**
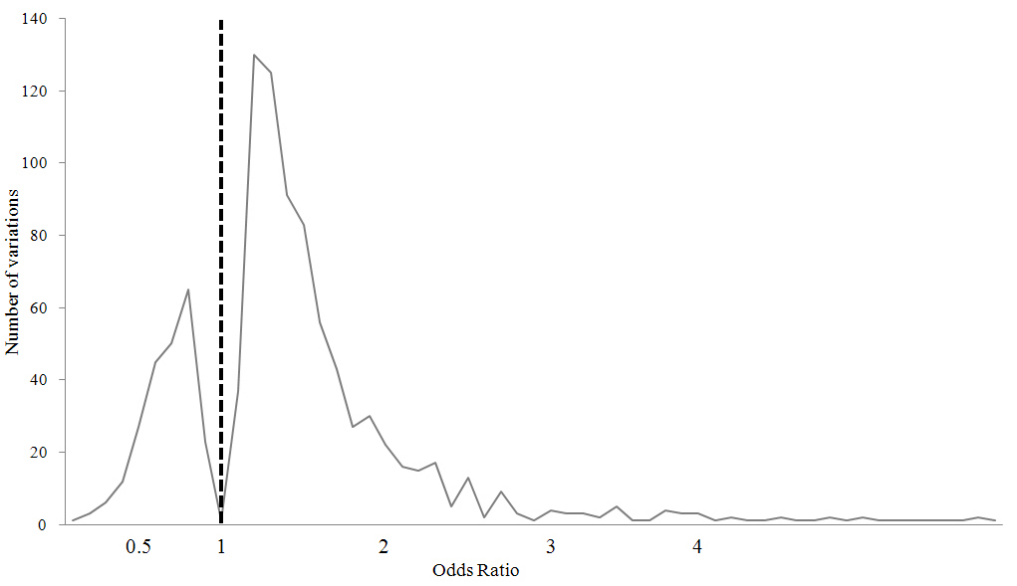
**Distribution of the OR for the association with T2D**.

Several statistical analyses were performed using these databases to characterize the published T2D association results. First, Figure 7 shows the distribution of the study ethnic populations used for the association with T2D. Mostly European and Asian populations were investigated with relatively few studies being carried out on Middle-East residents, American Indians, Africans and Latin Americans. Second, most of the genes listed in the database were published once and only 28.11% (149 genes from the total 530 genes) of genes were published more than twice (Figure 8). Figure 9 shows the distribution of a case sample size. More than 50% of publications used 100 ~ 500 participants as case samples. Most of the OR was distributed in the 0.5 ~ 2 range (Figure 10).

## Discussion

With the advances in technology for genetic studies, the amount of genetic information has increased over the last decade so that publications on the relationship between the disease and genetic variations have also increased explosively [8,9,11]. However, it might be difficult to summarize the huge data results in a systemic manner because information, such as the name of the SNP, gene and association result, was described differently in the papers. Several databases have been established to collect and present data to the user [4-7,12]. Nevertheless, it is difficult to find information of interest to the researchers because most databases are only archives. Researchers can search for informative data in public databases, for example, a literature search through a PubMed search, find disease-related genes using HuGE Navigator, and obtain GWAS publication data from the online GWAS summaries in NHGRI. However it is still difficult to compare all the data at a glance because the data is provided in text format only (refer table 1 for comparison).

**Table 1:** Comparison of T2D related DB with T2DGADB

	T2DGADB	T2D-DB^1^	Hindorff et al.^2^	Johnson et al.^3^	T1Dbase^4^
T2D Candidate genes	530	330	50	476	
Unique genes	421	NS	23	380	

T2D Candidate SNP Markers	2108	NS	76	6076	
Unique Markers	1874	NS	28	5841	

T2DM publication	701	8963	23	12	

coverage year	2000 ~ 2009. 10	~ 2009. 1	2005 ~ 2010. 10	2002. 12 ~ 2008. 2	~ 2010. 10

data coverage	T2D candidate gene approach, GWAS	T2D candidate gene approach, GWAS	GWAS (disease/trait)	GWAS (disease/trait)	T1D candidate gene approach, GWAS

association data format	text, graph	NA	text	text	text

feature	summary of T2D related genetic association study results(sample size, gene, SNP, MAF, p-value, OR, covariant, etc.)	summary of molecular factors using public databases (EST, Transcripts, Unigene, Homologene, GO, Pathways, Tissue Specific Expression, Protein-Protein interactor, Riskfactors, Complications)	summary of GWAS data (Disease, sample size, gene, SNP, MAF, p-value, OR, platform, CNV, etc.)	summary of GWAS data (Disease, sample size, gene, SNP, MAF, p-value, OR, platform, CNV, etc.)	summary of T1D related molecular factors (T1D susceptibility regions, Genetic data, Microarray data, functional annotation, Network & Pathway), analysis tools

1. T2D-Db: an integrated platform to study the molecular basis of Type 2 diabetes. Agrawal S et al. BMC Genomics. 2008 Jul 7;9:320.

2. Potential etiologic and functional implications of genome-wide association loci for human diseases and traits. Hindorff LA et al. Proc Natl Acad Sci USA. 2009 Jun 9;106(23):9362-7.

3. An open access database of genome-wide association results. Johnson AD et al. BMC Med Genet. 2009 Jan 22;10:6.

4. T1DBase: integration and presentation of complex data for type 1 diabetes research. Hulbert EM et al. Nucleic Acids Res. 2007 Jan;35(Database issue):D742-6.

This study implemented a database that stores the results of type 2 diabetes association study and detailed information on the study design, and provided graphs that represent the association results. The goal in developing T2DGADB was to provide researchers with quick and easy access to published T2D genetic association information. Moreover, the data in the database was manually curated by adding accuracy to it in order to help researchers evaluate the T2D association study results that have been published from 2001 to 2009. To use the data in T2DGADB, a scientist can begin with a gene with a published association with T2D, then find positive or negative results of all SNPs studied within the gene, discover OR, 95% CI and the p-value of each SNP through a box plot graph, and finally understand the study design information (e.g., study population, sample size) (Figure 1). This is a unique resource to show both study data on the candidate gene approach and GWAS data graphically for T2D researchers. The data can provide a starting point for a genetic study design, systematic review or reference search as well as produce primary genetic data for constructing a diabetes risk test in the preparation of personalized medicine.

This study is a small step in the preparation of a personalized diagnosis system. Using this data, T2D candidate genes can be selected and their risk be estimated. Eventually, the diabetes genetic association database can be utilized to make a computer program that provides health care providers with the individual susceptibility to diabetes for personalized medicine, and can be expanded to the selection of high risk groups for preventive medicine.

## Conclusions

In this study, a user-friend database of T2D genetic association of manually curated information was constructed. This database can be used for research purposes, such as an association and functional study of T2D related genes, and as a primary genetic resource to construct a diabetes risk test in the preparation of personalized medicine in the future.

## Availability and requirements

T2DGADB is freely available for academic and commercial users at http://t2db.khu.ac.kr:8080.

## Competing interests

The authors declare that they have no competing interests.

## Authors' contributions

JEL, KWH, HKP and BO conceptualised and designed the project. JEL, KWH, HSJ and YSK designed database scheme, input data structure and data curation condition. JEL and BO drafted the manuscript. YSK, HKP and BO contributed to the intellectual content of the manuscript. All authors read and approved the final manuscript.

## Pre-publication history

The pre-publication history for this paper can be accessed here:

http://www.biomedcentral.com/1472-6947/10/76/prepub
